# PDI is an essential redox-sensitive activator of PERK during the unfolded protein response (UPR)

**DOI:** 10.1038/cddis.2017.369

**Published:** 2017-08-10

**Authors:** Philip Kranz, Fabian Neumann, Alexandra Wolf, Fabian Classen, Mosche Pompsch, Tobias Ocklenburg, Jennifer Baumann, Kirsten Janke, Melanie Baumann, Kirsten Goepelt, Helena Riffkin, Eric Metzen, Ulf Brockmeier

**Affiliations:** 1 Institut für Physiologie, Universität Duisburg-Essen, Hufelandstraße 55, D45122 Essen, Germany

## Abstract

Endoplasmic reticulum (ER) stress leads to activation of the unfolded protein response (UPR) that results in transient suppression of protein translation to allow recovery but leads to cell death when stress cannot be resolved. Central to initiation of the UPR is the activation of the ER transmembrane kinase protein kinase R (PKR)-like endoplasmic reticulum kinase (PERK). Here we report that the thiol oxidoreductase ERp57 and protein disulfide isomerase-A1 (PDI), which belong to the same family of luminal ER oxidoreductases, have strikingly opposing roles in the regulation of PERK function. In HCT116 colon carcinoma cells, lentiviral depletion of ERp57 resulted in oxidation of PDI and activation of PERK, whereas depletion or chemical inhibition of PDI reduced PERK signaling and sensitized the cancer cells to hypoxia and ER stress. We conclude that oxidized PDI acts as a PERK activator, whereas ERp57 keeps PDI in a reduced state in the absence of ER stress. Thus, our study defines a new interface between metabolic redox signaling and PERK-dependent activation of the UPR and has the potential to influence future cancer therapies that target PERK signaling.

As an essential organelle in eukaryotic cells, the endoplasmic reticulum (ER) is the site of lipid and steroid synthesis and provides the major calcium reservoir for the cell. In addition, the ER is responsible for the correct folding of nascent membrane and export proteins during the early secretory pathway. Approximately 30% of all newly synthesized proteins pass this cell compartment.^1^ Importantly, protein folding capacity can be exceeded as a consequence of various stressful stimuli (e.g., energy deprivation, calcium depletion, hypoxia, viral infection).^2, 3^ The accumulating misfolded proteins clog the secretory pathway and become toxic for the cell.^4^ To prevent subsequent cell death, the stress-prone ER relies on a powerful cellular program to reestablish ER homeostasis that is termed the unfolded protein response (UPR). This complex signaling cascade includes three specific branches each of which possesses a distinct transmembrane receptor as a sensor for ER stress: the inositol-requiring enzyme 1 (IRE1), the activating transcription factor 6 (ATF6) and the protein kinase R (PKR)-like endoplasmic reticulum kinase (PERK).^5^ The UPR pursues the increase of the folding capacity via activation of IRE1 and ATF6 that leads to increased production of ER chaperones and, second, the decrease of the unfolded protein burden via PERK-dependent inhibition of translation initiation. In unstressed cells, the abundant ER-resident chaperone BiP/GRP78 binds to the luminal domain of all three receptors and keeps them in an inactive state. Upon ER stress, however, the accumulation of unfolded proteins leads to the dissociation of binding immunoglobulin protein (BiP) from the UPR sensors, thereby triggering either a single branch or full UPR activation depending on stress intensity.^6^

PERK, which is also known as the eukaryotic translation initiation factor 2-*α* kinase 3 (EIF2AK3), is a type I transmembrane ER receptor.^7^ The detachment of BiP from PERK during ER stress allows oligomerization and autophosphorylation of the cytoplasmic kinase domain.^8^ Once activated, PERK is capable of phosphorylating and thereby disabling its major substrate, the translation initiation factor-2*α* (eIF2*α*), leading to general inhibition of cap-dependent translation. At the same time, the expression of mRNA containing short upstream open reading frames is stimulated. An example of increased expression during stress is the activating transcription factor 4 (ATF4), a regulator of genes involved in the adaptation to ER stress. The particular relevance of the PERK branch is underlined by the identification of further direct PERK substrates. These include the transcription factors nuclear factor erythroid-2-related factor 2 (Nrf2), which is critical for redox homeostasis,^9^ and Forkhead/FOXO transcription factor 3 (FOXO3), which is involved in metabolic homeostasis.^10^ The second messenger molecule diacylglycerol (DAG) has also been shown to be a PERK substrate.^11^ As PERK activation is also linked to viral infections, neurodegenerative disorders (e.g., Alzheimer, Parkinson and prion disease)^12^ and, most notably, to cancer, there has been a growing interest in PERK as a potential clinical option for treatment of these diseases. Potent small-molecule PERK inhibitors were generated recently and tested in preclinical mouse studies on cancer and prion disease.^13, 14, 15^ However, while an extensive effort was made to elucidate the downstream signaling of PERK over the past years, much less is known about regulatory components upstream of PERK. Obviously, understanding the mechanisms of PERK regulation is mandatory to decide in which situations PERK inhibition could be beneficial. Actually, in other cases PERK activation might be the more reasonable option.^13^

Surprisingly few studies have shed light on processing and regulation of the ER stress sensor proteins themselves.^16, 17^ Of note, two recent studies reported new functions for members of the ER-resident protein disulfide isomerase family (PDIs, EC 5.3.4.1) in redox regulation of the UPR sensors through thiol–disulfide exchange: the first of these studies demonstrates that intramolecular disulfide bridges of the UPR sensor ATF6 have to be cleaved by PDIA5 for transport through the Golgi to its final destination in the nucleus.^18^ Second, Eletto *et al.*^19^ showed that PDIA6 is able to control inactivation of the UPR sensor IRE1alpha through direct binding via a disulfide bond in the luminal domain. The same report also presented first evidence of an interaction of PDIA6 with PERK. These studies sparked our interest in the redox regulation of PERK. In cultured human colon cancer cells we noticed that depletion of the thiol oxidoreductase ERp57, which is also termed PDIA3, exclusively activated the PERK branch of the UPR. Intriguingly, this effect was completely abolished by the double knockdown of PDI and ERp57. We took these results as a starting point to dissect the interplay between ERp57, PDI and the stress sensor protein PERK.

## Results

### Additional knockdown of PDI in HCT116 shERp57 cells reduces p53 stabilization and apoptotic signaling

**Figure 1:**
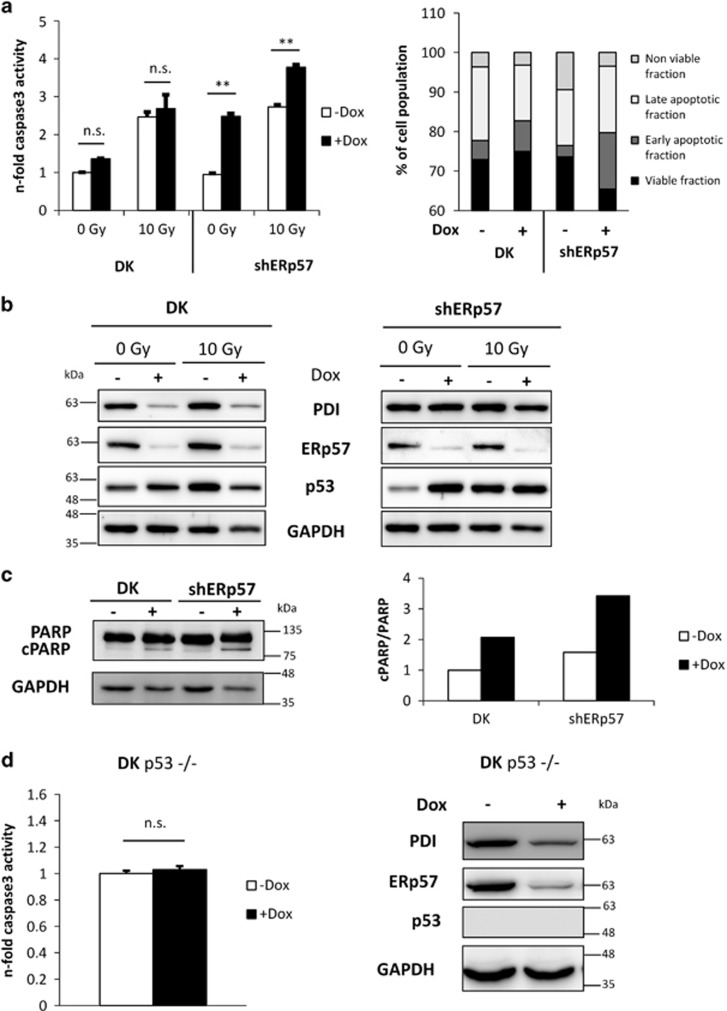
Simultaneous knockdown of ERp57 and PDI (DK) in HCT116 cells reduces apoptosis as compared with ERp57 knockdown alone. Every experiment was repeated twice and within the experiments at least three samples were treated in the same way. Bars represent mean±S.D. Two groups were analyzed by Student’s *t*-test, and more than two groups by ANOVA with *post hoc* Tukey’s test. Statistical significance is presented as **P<0.01. (**a**) At 96 h after KD induction and 48 h after irradiation with 10 Gy, caspase-3 activity was measured in whole-cell lysates of shERp57 and DK. Annexin/PI staining at 96 h after KD induction was used to validate the caspase-3 activity assays. (**b**) Representative western blots displaying KD efficiency of ERp57 and PDI and protein levels of p53. (**c**) At 96 h after KD induction, PARP cleavage of DK and shERp57 cells was shown by western blot. (**d**) Apoptosis levels in DK p53−/− cells measured by caspase-3 activity assay. Representative western blot shows KD efficiency and p53 deficiency in p53−/− cells

In a previous study we demonstrated induction of p53-dependent apoptosis upon depletion of ERp57 in HCT116 cells that showed additive knockdown (KD) effects under combined treatment with ionizing radiation and chemotherapeutic agents. Using a doxycycline-inducible lentiviral shRNA approach, the current data showed that additional KD of the ER oxidoreductase PDI (combined shERp57/shPDI KD; afterwards referred to as double knockdown (DK)) significantly reduced caspase-3 activity in HCT116 cells. When PDI and ERp57 were depleted simultaneously in contrast to ERp57 KD alone, combination with radiation did not elevate the apoptotic response (Figure 1a). Western blots revealed ∼90% KD efficacy with respect to both ER oxidoreductases, but a less pronounced induction of the tumor suppressor protein p53 when ERp57 and PDI were knocked down at the same time (Figure 1b). Caspase-3-dependent cleavage of poly-(ADP ribose) polymerase 1 (PARP) as well as Annexin/PI staining confirmed these observations (Figures 1a and c). To assess whether the less pronounced apoptotic signaling in DK cells is still p53 dependent, an isogenic HCT116 p53-negative cell line was used. While equal KD efficiency was achieved in both cell lines, caspase-3 activation could not be observed upon DK in p53-negative cells (Figure 1d).

### Cell viability and clonogenic survival are reduced upon depletion of ERp57 and PDI

**Figure 2:**
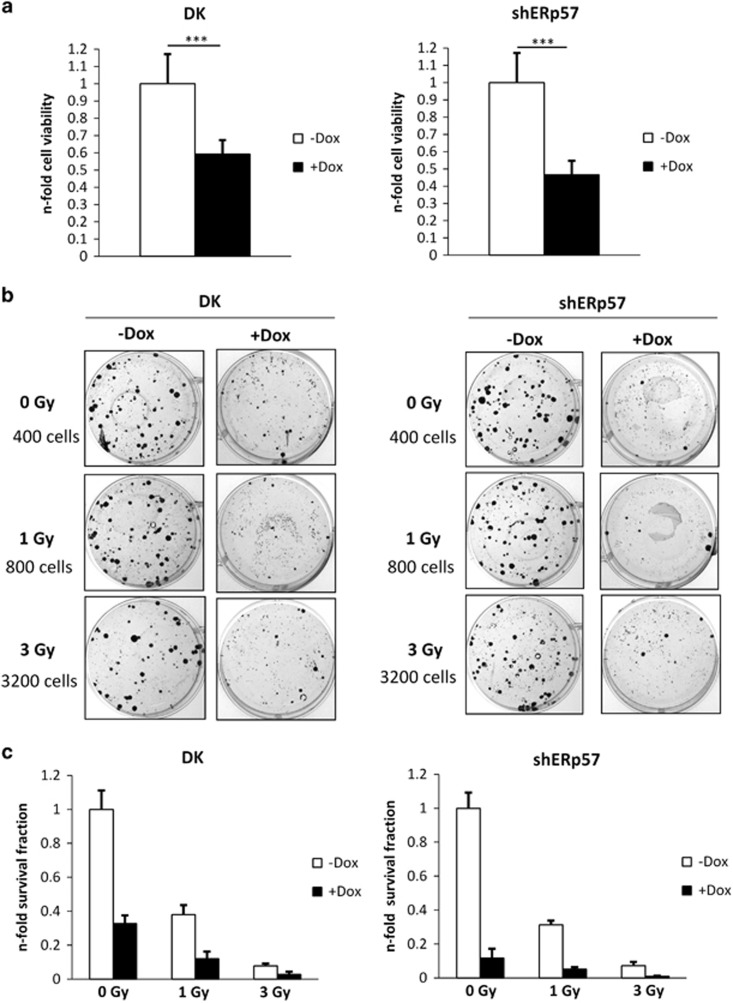
Cell viability and clonogenic survival are reduced in HCT116 shERp57 and DK. Every experiment was repeated twice and within the experiments at least three samples were treated in the same way. Bars represent mean±S.D. (**a**) MTT viability assay was performed 96 h after KD induction in DK and shERp57 cells. Statistical significance is presented as ***P<0.001. (**b**) Colony formation in a clonogenic survival assay after 1 or 3 Gy radiation combined with DK or shERp57 cells. (**c**) Quantitative display of the data set from (**b**)

Although apoptosis induction was impaired in DK cells, cell viability was reduced as measured by MTT assays (Figure 2a). Knockdown of shERp57 alone showed the most prominent reduction of viability with a loss of 57%, whereas the decrease in DK cells is less drastic with 40% respectively that is in line with a smaller apoptotic cell fraction. Clonogenic survival in colony formation assays of both cell lines was strongly reduced through KD induction alone, but even more pronounced in shERp57 cells (Figure 2b). DK reduced clonogenic survival to 30%, comparable to 1 Gray (Gy) irradiation, whereas ERp57 depletion alone diminished the survival fraction (SF) almost as effectively as 3 Gy irradiation (9% SF). Consistent effects upon combination of KD and ionizing radiation were obtained for both cell lines, although with a higher efficacy through ERp57 depletion alone (Figure 2b).

### IRE1, ATF6 and PERK are not activated in DK cells after combined depletion of ERp57 and PDI

**Figure 3:**
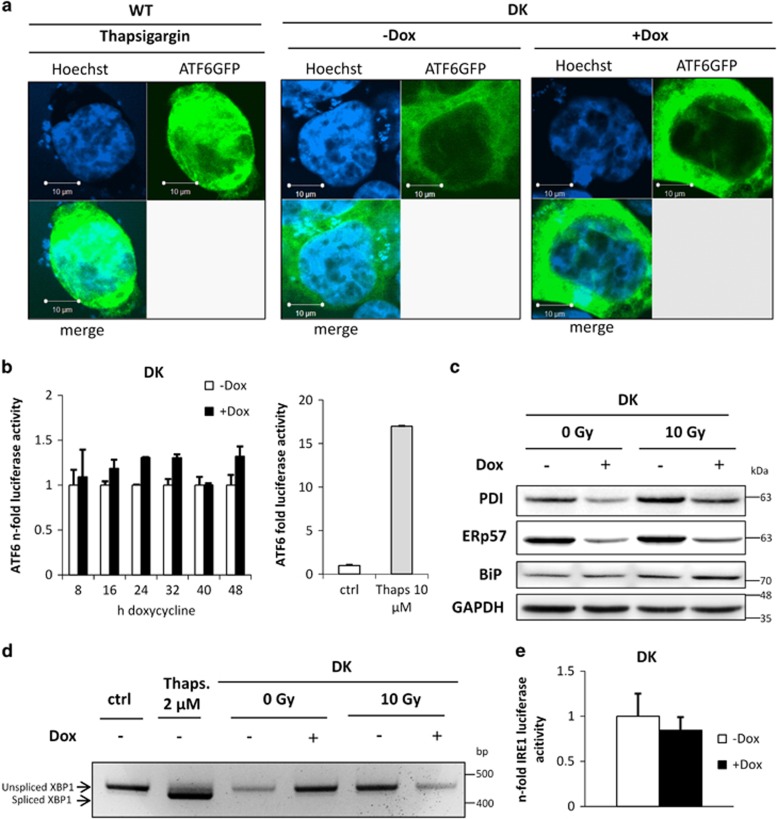
Simultaneous depletion of ERp57 and PDI does not activate the UPR sensors ATF6 and IRE1. Every experiment was repeated twice and within the experiments at least three samples were treated in the same way. Bars represent mean±S.D. (**a**) Confocal microscopy images of HCT116 WT or DK cells transfected with an ATF6-GFP fusion construct. The 2 *μ*M thapsigargin (Thaps) was used as positive control. (**b**) ATF6 reporter gene assay, 10 *μ*M Thaps was used as positive control. (**c**) Western blot showing KD efficiency and the ATF6 target gene BiP. (**d**) At 48 h after irradiation and 96 h after KD induction, RT-PCR was performed to detect the spliced form of XBP1 as an indicator for IRE1 activation. (**e**) XBP1 reporter gene assay 96 h after KD induction

**Figure 4:**
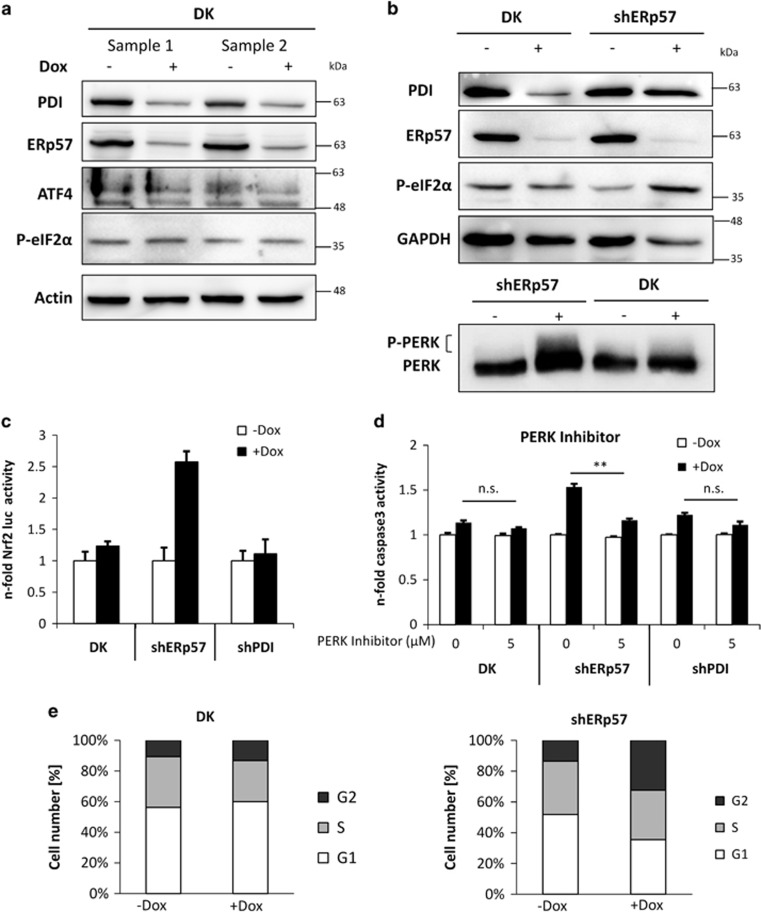
The UPR sensor PERK is activated in HCT116 shERp57 cells and contributes to apoptosis induction. KD was induced for 96 h. Every experiment was repeated twice and within the experiments at least three samples were treated in the same way. Bars represent mean±S.D. Statistical significance is presented as **P<0.01. (**a** and **b**) Comparison of PERK phosphorylation, eIF2*α* phosphorylation and ATF4 expression in shERp57 and DK cells by western blot. (**c**) Luciferase assay performed with an Nrf2-responsive element plasmid in DK, shERp57 and shPDI cells. (**d**) Caspase-3 activity assay in shERp57, shPDI and DK cells after 48 h of PERK inhibition with GSK2606414. (**e**) HCT116 shERp57 und DK were irradiated with 10 Gy 48 h after KD induction. After 48 h, cell cycle distribution was determined by PI staining and subsequent FACS analysis

Considering that both proteins, ERp57 and PDI, are involved in important biological processes,^20^ a higher survival probability for DK cells appears surprising, although their growth and viability is still impaired massively. However, the ER is known to cope with stressful conditions through activating the UPR branches ATF6, IRE1 and PERK. A possible explanation for less pronounced apoptosis and enhanced survival caused by the DK as compared with the ERp57 KD was a potential increase in UPR coping mechanisms. Therefore, these pathways were checked using fluorescence microscopy, western blot analysis, luciferase reporter gene assay and PCR. Figure 3a shows fluorescence microscopy pictures of HCT116 wild-type (WT) cells and DK cells transfected with ATF6-GFP plasmid. In WT cells, induction of ER stress by thapsigargin resulted in a massive migration of ATF6 to the nucleus. However, after 72 h of DK induction there was no obvious translocation into the nucleus. To confirm these results, luciferase assays with a plasmid containing an ATF6-responsive element were performed at different time points after doxycycline exposure (Figure 3b). The induction of DK only caused a marginal activation of luciferase activity as compared with an almost 18-fold induction after treatment with the ER stress inducer thapsigargin. In addition, we were unable to detect any upregulation of the ATF6 target gene BiP on the protein level after DK induction, whereas irradiation induced BiP (Figure 3c). Next, we examined RNase activity of IRE1 that results in cleavage of the X-box binding protein-1 (XBP1) mRNA and a 26 bp shift in agarose gel electrophoresis (Figure 3d). When thapsigargin was used as positive control, a migration shift was clearly visible. After DK induction, even in combination with an irradiation dose of 10 Gy radiation, a shift was not detectable, indicating the inactive state of IRE1. Furthermore, reporter gene assays using pFLAG-XBP1u-FLuc did not reveal any transcriptional activity of XBP1 after 96 h of DK, confirming the XBP1 splicing results (Figure 3e). PERK phosphorylation and activation of its downstream targets eIF2*α* and ATF4 were examined via Western blotting (Figures 4a and b). Upon DK, an increase of phosphorylated eIF2*α* or ATF4 was not detected, thus demonstrating an inactive status of PERK as shown for ATF6 and IRE1 before. Comparing DK with shERp57 cells showed that PERK signaling through additional KD of PDI was abrogated as measured by phosphorylation of PERK and eIF2*α* by western blotting (Figure 4b). Another well-documented target for PERK is Nrf2. Therefore, we assessed PERK activity by means of an Nrf2-responsive luciferase reporter gene assay.^21^ While an ERp57 knockdown substantially activated the reporter, DK as well as PDI knockdown did not have a significant effect (Figure 4c). In a previous study, we had described a PERK-dependent proapoptotic signaling pathway activated by KD of ERp57 that could be blocked by the selective PERK inhibitor GSK2606414.^22^ In the present study, apoptosis was significantly reduced in shERp57 through PERK inhibition, almost exactly down to the level of DK cells. In DK and shPDI cells, chemical inhibition of PERK had no effect, reinforcing the observation that PERK is inactive when PDI is depleted alone or in combination with ERp57 (Figure 4d). Furthermore, we compared cell cycle progression in DK and shERP57 cells. Notably, the enhanced G2-M arrest that occurred after ERp57 depletion^22^ vanished after additional PDI KD (Figure 4e).

### Functional PDI is required for sufficient PERK activation and is kept by ERp57 in a reduced state

**Figure 5:**
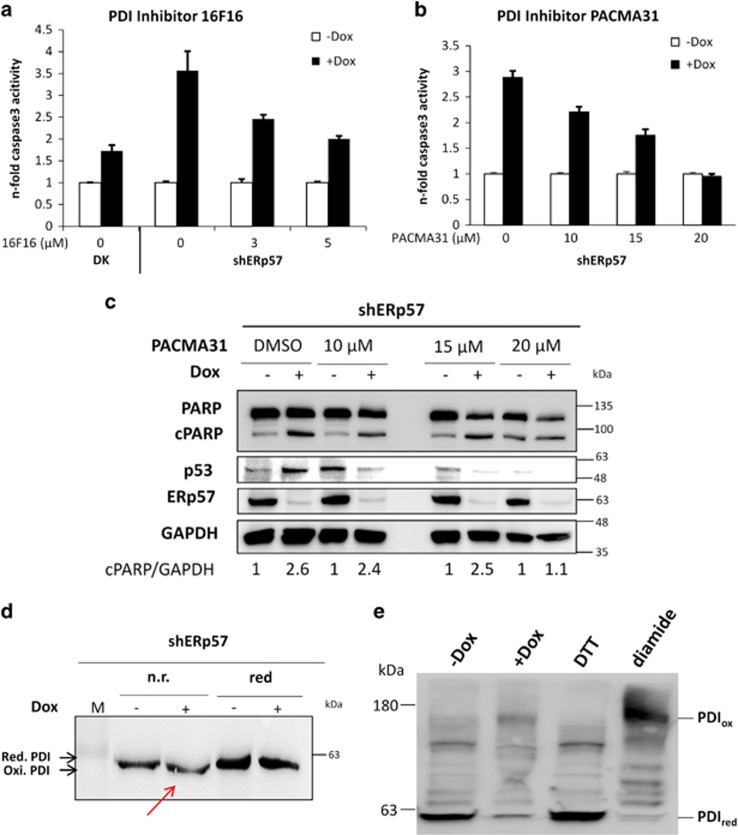
Functional PDI is required for sufficient induction of apoptotic PERK signaling and is kept in a reduced state by ERp57. Every experiment was repeated twice and within the experiments at least three samples were treated in the same way. Bars represent mean±S.D. (**a**) Caspase-3 activity assay of shERp57 cells 96 h after KD induction and 24 h incubation with the PDI inhibitor 16F16. (**b**) HCT116 shERp57 cells were treated with various concentrations of the PDI inhibitor PACMA31 for 24 h after 72 h of KD induction and were used for caspase-3 activity assay. (**c**) Corresponding western blots of shERp57 cells. (**d**) At 96 h after ERp57 KD induction, nonreducing SDS-PAGE and subsequent western blot was performed with whole-cell lysates. (**e**) Redox-dependent mobility shift of PDI after mPEG-mal alkylation. At 96 h after ERp57 KD induction, shERp57 cells were subjected to the mPEG-mal alkylation protocol followed by SDS-PAGE and western blot. Oxidized cysteines of PDI were alkylated with the mPEG-mal_5000_ polymere, leading to an increased shift in gel migration. DTT and diamide were used as controls for fully reduced (PDI_red_) and fully oxidized (PDI_ox_) forms of PDI

To test whether PDI is required in a functional or just in a structural state for the activation of PERK and p53, PDI was blocked chemically in shERp57 cells using 16F16^23^ or PACMA31.^24^ In caspase-3 assay, both inhibitors led to a dose-dependent decrease of apoptosis in shERp57 cells when PDI was functionally inhibited, mimicking the results generated after lentiviral depletion of PDI (Figure 5a, compare Figure 4d). Western blot analysis showed less PARP cleavage and reduced p53 protein levels using PACMA31 in combination with ERp57 KD (Figure 5c). Functional PDI contains four active site cysteine residues that can exist in an oxidized or reduced state. To measure differences in the redox state of these cysteines, nonreducing SDS-PAGE was performed. Remarkably, PDI mainly appeared to be reduced in control cells but was predominantly oxidized following ERp57 depletion (Figure 5d). To confirm this result, an additional PEG-maleimide modification assay was performed. Within this assay disulphide bonds, that is, the oxidized state, undergo alkylation that shifts electrophoretic mobility to an apparently higher molecular weight, whereas thiol groups, that is, the reduced state, remain without alkylation. These experiments demonstrated a switch from the reduced to the oxidized form of PDI after KD of ERp57 (Figure 5e). Collectively, these data demonstrate that ERp57 acts as a reductase for PDI in cells and, importantly, that accumulation of oxidized PDI induces proapoptotic PERK signaling.

### Role of PDI for PERK activation under global ER stress

**Figure 6:**
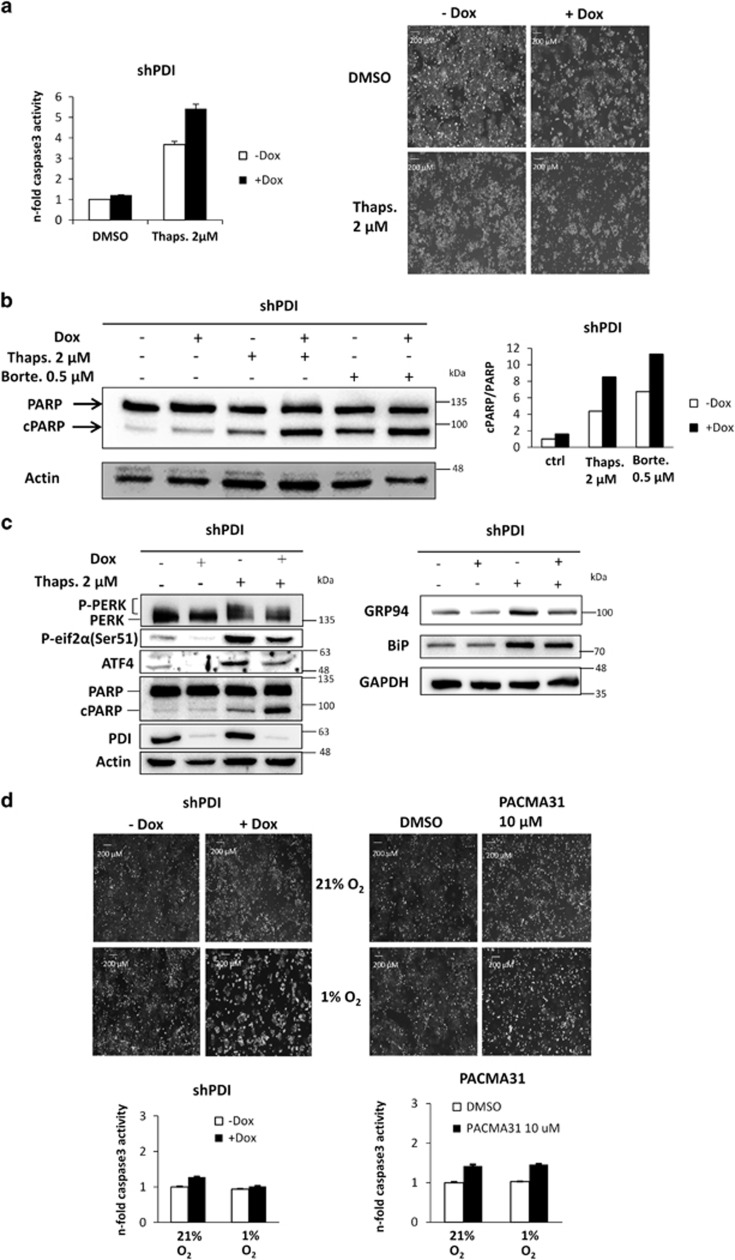
PDI is required for sufficient and cytoprotective PERK activation under global ER stress. Every experiment was repeated twice and within the experiments at least three samples were treated in the same way. Bars represent mean±S.D. (**a**) HCT116 shPDI cells were treated with 2 *μ*M thapsigargin for 20 h and used for caspase-3 assay. Corresponding phase contrast microscopy pictures are shown. (**b**) Western blot shows PARP cleavage after 24 h of treatment with thapsigargin (2 *μ*M) or bortezomib (0.5 *μ*M) and 96 h KD in shPDI cells. (**c**) Western blot of shPDI cells treated with 2 *μ*M thapsigargin. PERK, P-eIF2*α*, ATF4 GRP94, PARP, BiP and PDI are shown. (**d**) shPDI- or PACMA31-treated HCT116 cells were cultured under 21% or 1% oxygen. Phase contrast microscopy and caspase-3 assays are shown

PERK activation and the ensuing phosphorylation of eIF2*α* as well as ATF4 induction and its downstream transcriptional activity were described as cytoprotective and mechanisms that favor survival under global ER stress by limiting the protein amount in the ER and by increasing the folding capacity through transcriptional upregulation of ER chaperones. As described above, upon depletion of ERp57, PDI accumulates in an oxidized state, followed by PERK activation and proapoptotic signaling through p53. To verify that PDI also plays a cytoprotective role during global ER stress, HCT116 shPDI cells were used as this cell line displayed only a mild phenotype upon KD induction (Figure 4). Of note, treatment with thapsigargin led to a more severe apoptosis rate when combined with PDI KD (Figure 6a). The inhibition of PERK activation through PDI KD resulted in a higher vulnerability against the ER stressors thapsigargin and bortezomib, as measured by PARP cleavage (Figure 6b). Administration of thapsigargin to shPDI cells neither induced PERK phosphorylation nor upregulated its specific downstream targets ATF4 and GRP94 that demonstrates a lack of PERK signaling (Figure 6c). PERK activation and its downstream signaling have been shown to be mandatory for hypoxic adaptation of different cancer and noncancer cell lines through upregulation of ROS defense pathways and glutamine metabolism.^25^ To assess whether depletion or chemical inhibition of PDI impairs this adaptation processes similar to PERK blockade, proliferation was examined when the cells were cultured under hypoxic conditions (1% O_2_). Cells in which PDI was depleted or chemically inhibited displayed massively reduced proliferation upon oxygen deprivation as shown by brightfield microscopy (Figure 6d). Apoptosis was only marginally induced by oxygen deprivation and PDI KD, alone or in combination, and therefore could not explain the drastic drop in cell numbers. Rather, the loss of hypoxic adaptation in the absence of PDI points to an inactive PERK branch.

## Discussion

**Figure 7:**
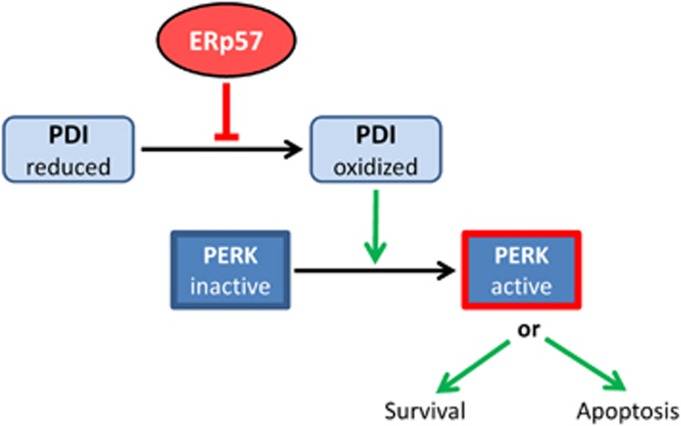
A schematic representation of the redox-sensitive events occurring in the lumen of the endoplasmic reticulum upstream of PERK activation. The oxidoreductase ERp57 blocks oxidation of PDI. Thus, loss of functional ERp57 results in PDI oxidation that is in turn necessary for the activation of PERK. Operation of the switch by which active PERK either leads to survival or to apoptosis is not understood so far

The ER stress sensor protein kinase PERK has attracted a substantial amount of interest for several reasons: as a prosurvival factor, it helps to adapt cellular metabolism to environmental cues such as oxygen or amino-acid deprivation. These situations develop in a number of highly prevalent diseases such as neurodegenerative processes or tumor development and growth.^26^ Furthermore, we and others showed that PERK is also implicated in cell death decisions because after an initial adaptation period in which protein translation is reduced, the activation of PERK can result in p53-dependent or -independent apoptosis.^22, 27, 28^ Recent work also indicated that PERK is directly involved in the switch between apoptosis and autophagy via modulation of p38.^29^ Nevertheless, it must be emphasized that neither upstream events, that is, regulation of PERK nor operation of the switch from cell survival to apoptosis induction are completely understood presently. In our initial experiments we were surprised to see that the UPR- and apoptosis-related phenotype that we observed after single KD of ERp57 was almost completely abrogated after the additional depletion of PDI. This was remarkable as both homologous enzymes are well known for their cell protective contributions in oxidative folding and ER quality control. In fact, the true relevance of PDI for the PERK branch of the UPR was not exposed until we tested the ER stress response after single KD or chemical inhibition of PDI. We noticed that only functional PDI was able to trigger PERK activity beyond basal levels and is therefore indispensable for a proper PERK-related stress response. It was reported elsewhere that five different PDI inhibitors were found to block apoptosis after accumulation of misfolded proteins in a PC12 cell-based model for Huntington disease.^23^ Furthermore, a downregulation of PDI in mouse embryonic fibroblasts reduced apoptosis upon treatment with different ER stress inducers.^30^ Therefore, our discovery is probably not restricted to colon carcinoma cell lines but rather displays a general control mechanism of PERK activation. We further extended the underlying mechanism of PDI-related PERK activation through the involvement of ERp57, that is, a second, closely related oxidoreductase as depicted in Figure 7. Herein, we propose a redox-regulated model of PERK activation where under nonstressed conditions ERp57 keeps PDI in a reduced state that prevents PERK activation. In the absence of ERp57, however, PDI accumulates in an oxidized form that allows oligomerization and activation of PERK. The occurrence of a predominantly reduced form of PDI in unstressed cells is supported by several studies that examined the redox state of PDI.^31, 32, 33, 34^ Although not addressed directly in our current study, ERp57 has been reported to form a complex with PDI.^35^ In addition, a disulfide exchange *in vitro* between PDI and ERp57 has been demonstrated earlier. It was postulated that ERp57 undergoes oxidation by PDI,^36^ in line with our hypothesis of ERp57 acting as a reductase for PDI.

The ER thiol oxidoreductase PDIA6 was recently identified as a negative regulator of both UPR sensors IRE1 and PERK.^19^ The authors stated that PDIA6 binds directly to their luminal domain in a cysteine-dependent manner. As it is plausible to postulate a similar mode of action for PDI, we performed numerous co-immunoprecipitation experiments to demonstrate a direct interaction between PERK and PDI. However, although we saw a weak interaction in some of the experiments using endogenous proteins, the results were not consistent and were not improved when both proteins were overexpressed. In consequence, our data collectively argue for a very transient interaction or an indirect mechanism of PDI-dependent activation of PERK signaling. Because of the potential involvement of disulfide bridges within the luminal domain of PERK in the activation reaction, the role of the four luminal cysteines of PERK for protein interaction with PDI and UPR signaling is the subject of an ongoing investigation.

Previously, we localized ERp57 outside the ER in the cytosol and found evidence for its role in growth-related pathways.^22^ In the current study, we observed a similar dramatic cell growth inhibition after combined depletion of PDI and ERp57. The mild phenotype found after single KD of PDI suggested initially that ERp57 alone promotes proliferation in tumor cells. However, global ER stress, as induced by hypoxia for example, revealed the paramount importance of PDI for PERK-dependent adaptation of cancer cells to harsh environmental conditions. Previous reports by other authors strongly suggest that the cellular contributions of PDI to cancer growth is not limited to PERK activation as PDI appears to be present in the nucleus, cytosol and on the cell surface.^20^

With respect to medical relevance of the UPR, PERK modulators certainly have potential in pharmacology and, indeed, newly developed PERK inhibitors displayed excellent antitumoral as well as neuroprotective effects in preclinical studies.^15, 14, 37, 38^ Nevertheless, their therapeutic success is still questionable as a defect in PERK function causes severe pancreatic degradation^39^ and contributes to the human Wolcott–Rallison syndrome, a disorder characterized by diabetes mellitus and growth retardation.^40^ In fact, the PERK inhibitor GSK2656157 caused severe damage to pancreatic cells in a xenograft tumor model.^15^ Notably, Yu *et al.*^41^ proposed an additional anti-interferon treatment during administration of PERK inhibitors to bypass their pancreatic toxicity. Our data suggest instead that common PDI inhibitors provide an efficient but potentially less toxic way to block PERK for cancer treatment. This proposal would be in line with a recent report, where the PDI inhibitor PACMA 31 significantly suppressed ovarian tumor growth while it was substantially less toxic to normal tissue.^24^ Certainly, it would be informative to test whether PACMA31-mediated inhibition of PDI influences PERK signaling in these authors’ model. Although the molecular mechanism of PDI-mediated regulation of PERK is elusive at this point, it is noteworthy that PDI acts on the luminal domain of PERK, whereas PERK inhibitors target the cytoplasmic kinase domain with high efficiency. It is conceivable that compared with a total PERK inhibition, PDI depletion leads to a moderate downregulation of PERK activity that could on one side prevent tumor cells from adapting to nutrient deficiency or hypoxia but would favor survival of normal tissue at the same time.

## Materials and methods

### Antibodies and reagents

All chemical compounds were purchased from Sigma (Munich, Germany) except the PERK inhibitor GSK2656157 that was from Millipore (Billerica, MA, USA) and bortezomib (PS-341) from UBPBio (Göttingen, Germany). Antibodies against PERK, GRP94, P-eIF2*α*, BiP and ATF4 were from Cell Signalling (Frankfurt/Main, Germany), anti-actin and anti-GAPDH from Sigma, anti-PDI from R&D Systems (Minneapolis, MN, USA) and anti-p53 from Millipore (Billerica, MA, USA). The ERp57 antibody was purchased from Abcam (Cambridge, UK). HRP-coupled secondary anti-mouse and anti-rabbit antibodies were from Dako (Hamburg, Germany).

### Cell culture, transfection and lentiviral transduction

HCT116 p53+/+ and p53−/− cells were cultured in McCoy’s 5 A medium (Lonza, Basel, Switzerland), HEK293T cells for lentivirus production were cultured in DMEM High Glucose (Invitrogen, Darmstadt, Germany). Cell culture media were supplemented with 10% FBS and penicillin/streptomycin. Viafect (Promega, Mannheim, Germany) was used for transient transfection of cell cultures in a ratio of 3 : 1 (*μ*l reagent per *μ*g DNA) as suggested in the manufacturer’s protocol. Lentiviral particles were produced in HEK293T cells.^42^ Briefly, 2 × 10^5^ cells were incubated for 24 h with 2 × 10^6^ lentiviral particles together with 8 *μ*g/*μ*l polybrene for better adherence. After transduction, the cells were selected by puromycin incubation. For generation of double KD cells, 2 × 10^5^ previously infected cells were again incubated for 24 h with lentiviral particles containing the second shRNA. Selection was not performed after the second lentiviral transduction. For knockdown experiments KD was induced by addition of 250 ng/ml doxycycline.

### Caspase-3 enzyme activity assay

To quantify apoptotic signaling caspase-3 activity was measured by cleavage of the acetyl Asp-Glu-Val-Asp 7-amido-4-methylcoumarin (Ac-DEVD-AMC; A1086, Sigma) as described previously.^43^ Cleavage releases fluorescent 7-amino-4-methylcoumarin (AMC) that was quantified every 10 min over 4 h in a fluorescence reader (Synergy HT, Biotek, Bad Friedrichshall, Germany). Single time point measurements in the linear range of the reaction were used and presented in bar graphs. For validation of caspase-3 activity PARP cleavage was evaluated by western blotting.

### Annexin/propidium iodide (Anx/PI) staining

Anx/PI staining was performed as described previously.^22^ In brief, cells were cultured and treated as indicated. 1 × 10^5^ cells were incubated with 80 *μ*g/ml PI and 9.6 *μ*g/ml Annexin V conjugated to Pacific Blue (No. 640917, Biolegend, San Diego, CA, USA) in Annexin V binding buffer (No. 422201, Biolegend) for 15 min at room temperature. Analysis was performed on a FACS Canto II (BD Biosciences, Franklin Lakes, NJ, USA). A total of 10 000 cells per sample were analyzed. For data analysis FCS Express 4 Flow software was used (De Novo Software, Los Angeles, CA, USA).

### Cell viability and clonogenic survival assay

For MTT cell viability assays 2000 cells were plated in 96-well plates. Knockdown was induced and cells were incubated for 96 h. Afterwards cells were exposed to 0.5 mg/ml 3-(4,5-dimethylthiazol-2-yl)-2,5-diphenyltetrazolium bromide for 3 h and lysed in MTT lysis buffer containing 99.4% DMSO and 10% SDS. Absorption was measured at 540 nm in a fluorescence reader (Synergy HT, Biotek). The results were plotted in bar graphs indicating viability with control samples standardized to 1.

To measure clonogenic survival, 100 to 6400 cells were plated in collagen-1-coated 6-well plates and the KD was induced. After 16 h of incubation, the cells were irradiated with either 1 or 3 Gy followed by 10–14 days of incubation. At the end point of the experiment, colonies were washed once with PBS, fixed with 0.4% PFA and 70% ethanol, stained with Coomassie brilliant blue (0.1 Coomassie blue, 5% acetic acid, 45% methanol) and were photographed with an FX7 documentation system (Peqlab, Erlangen, Germany). Colonies containing >50 cells were counted. The plating efficacy (PE) and the survival fraction (SF) were calculated with the formulas ‘PE=number of colonies formed/number of cells seeded’ and ‘SF=number of colonies formed/number of cells seeded × PE’, respectively.

### Fluorescence microscopy

5 × 10^4^ cells were plated on collagen-1-coated glass cover slides in a 24-well plate and transfected with 1 *μ*g pEGFP-ATF6 as described.^44^ After 4 h, the transfection medium was removed and the KD induced. After 72 h, the medium was removed, cells were fixed with 0.4% PFA in PBS and counterstained with 3.3 *μ*g/ml HOECHST32444. Translocation of ATF6-GFP was visualized using a Zeiss LSM510 inverse confocal microscope with a 63 × /1.2 NA oil immersion lens (Carl Zeiss, Heidelberg, Germany).

### Plasmids and shRNA sequences

For transient transfections the following plasmids were used: pGL3-8xARE (Wang *et al.*^21^), p5xATF6-GL3 (#11976, Addgene, Cambridge, MA, USA), pEGFP-ATF6 (#32955, Addgene), pFLAG-XBP1u-FLuc (31239, Addgene), p5xATF6-GL3 (#11976, Addgene), pGL4.74 (#E6921, Promega) and pCMMP-dnPERK-IRES-eGFP (aaΔ592-1061=ΔC PERK) (#36954, Addgene). For the production of lentiviral particles in HEK293T, the cells were co-transfected with pMD2.G (#12259, Addgene), psPAX2 (12260, Addgene) and Tet-pLKO-puro (#21915, Addgene) or pWPXL (#12257, Addgene) (expressing GFP for transfection control). Knockdown cell lines were generated by using the following shRNA sequence against ERp57 5′-GGAATAGTCCCATTAGCAAAG-3′ (corresponding to 374–394 bp of human ERp57 mRNA, GenBank acc. no. NM_005313) and PDI 5′-GTGTGGTCACTGCAAACAGTT-3′ (corresponding to 1385–1405 bp of human PDI mRNA, GenBank acc. no. NM_000918).

### PCR for XBP1 splicing

Total RNA was isolated using the Qiagen RNeasy mini kit (Hilden, Germany). Reverse transcription for mRNA analysis was performed with the iScript Select cDNA synthesis kit (Bio-Rad, Hercules, CA, USA). The samples were used to detect spliced and unspliced XBP1 cDNA using the forward primer 5′-AAACAGAGTAGCAGCTCAGACTGC-3′ and the reverse primer 5′-CCTTCTGGGTAGACCTCTGGGAG-3′. The PCR products were loaded on a 2.5% agarose gel and separated to visualize a 26 bp shift.

### Luciferase reporter gene assay

5 × 10^4^ cells were seeded in 24-well plates and transfected with 250 or 500 ng of one of the reporter plasmids pGL3-8xARE or p5xATF6-GL3. In both reporter assays, 100 ng of the control plasmid pGL4.74 (which expresses *Renilla* luciferase) was co-transfected to normalize for transfection efficiency. To assess XBP1 transcriptional activity, the cells were transfected with 500 ng pFLAG-XBP1u-FLuc. The transfections were performed with ViaFect (Promega) following the manufacturer’s instructions. Luminescent signals were generated with the Dual-Luciferase Reporter Assay (Promega). Luciferase activity was measured using a GloMax luminometer (Promega). Firefly luciferase activity was normalized to *Renilla* luciferase to eliminate differences in transfection efficacy.

### Polyethylene glycol maleimide (PEG-mal) alkylation

To better distinguish between the oxidized and the reduced form of PDI, a modified *N*-ethylmaleimide (NEM) alkylation assay^31^ using methoxy polyethylene glycol 5000 maleimide (mPEG-mal_5000_, Sigma) was performed as follows. After seeding HCT116-shERp57 cells in 6-well plates, ERp57 KD was induced and the cells were grown for 96 h. In control dishes, cells were incubated for 15 min with either 10 mM DTT (AppliChem, Darmstadt, Germany) to fully reduce or 5 mM diamide (Sigma) to fully oxidize PDI. The growth medium was removed and the cells were incubated for 20 min on ice in PBS containing 20 mM NEM (Sigma) that binds to SH-groups and makes them inaccessible for further modification. After washing with PBS, the cells were lysed in RIPA buffer (50 mM Tris pH 7.5, 2 mM EDTA, 150 mM NaCl, 1% Nonidet P40, 0.1% SDS, 0.5% sodium desoxycholate and protease/phosphatase inhibitor cocktail (#5872, Cell Signaling, ZA Leiden, The Netherlands)). Next, 12 mM TCEP solution (Thermo Scientific, Darmstadt, Germany) was added to completely reduce the remaining disulfide bonds. After 20 min at RT, the reduced thiols were alkylated during incubation with 15 mM mPEG-mal_5000_ for 60 min at RT. After adding SDS sample buffer, lysates were boiled for 5 min and used for SDS-PAGE and further western blot.

### SDS-PAGE and western blotting

For reducing conditions, samples were prepared in RIPA lysis buffer. For nonreducing conditions caspase-3 lysis buffer (Tris (pH 7.3) 50 mM, NaCl 150 mM, Nonidet P40 1%) and SDS-sample buffer without DTT/TCEP and *β*-mercaptoethanol were used. Proteins were separated using a 7.5–10% polyacrylamid gel and afterwards transferred onto a PVDF membrane. Unspecific binding sites were blocked using 5% skimmed milk in TBS-T (50 mM Tris/HCl, 150 mM NaCl, 0.5% Tween-20, pH 7.2). Antibody incubations were performed as recommended by the manufacturer. For detection of HRP-coupled secondary antibodies, an ECL kit (34095, Thermo Fisher Scientific) and an FX7 chemoluminescence documentation system (Peqlab) were used.

### Statistical analysis

All results were obtained in at least three independent experiments each of which was performed in triplicate. In bar graphs, mean plus S.D. of three independent samples of a representative experiment is given. For statistical analysis between two groups, Student’s *t*-test was used. When more than two groups from one experiment were tested, ANOVA was applied with *post hoc* Tukey’s test. Significance is presented as *P<0.05, **P<0.01 and ***P<0.001.
